# Do medical students like communication? Validation of the German CSAS (Communication Skills Attitude Scale)

**DOI:** 10.3205/zma000953

**Published:** 2015-02-11

**Authors:** Anne-Kathrin Busch, Katrin Rockenbauch, Gabriele Schmutzer, Elmar Brähler

**Affiliations:** 1 University Hospital Leipzig, Department of Psychic Health, Division for Medical Psychology and Medical Sociology, Leipzig, Germany

**Keywords:** communication, medical education, undergraduate, attitude of health personnel

## Abstract

**Objectives: **Attitudes towards communication skills of medical undergraduates can be gathered using the Communication Skills Attitude Scale (CSAS). We aimed to develop a German version of the CSAS (CSAS-G) in order to explore attitudes towards communication skills in a German cohort. Additionally the potential influence of demographic factors was examined.

**Methods: **We realized the CSAS-G and conducted a survey with 529 participants from 3 different years of study. We then carried out an explorative as well as confirmatory factor analysis and compared the attitudinal scores. Multiple regression analysis was performed.

**Results: **The confirmatory analysis confirmed the two-subscale system revealed by the explorative factor analysis. Students indicate low levels of negative attitudes and moderate levels of positive attitudes. Attitudinal scores differ significantly in relation to gender.

**Conclusion: **The CSAS-G can be used in German cohorts to evaluate attitudes towards communication skills. Medical students in our study show basically a positive approach. Further investigation is necessary to explore and understand attitudes towards communication skills of German medical students.

## Zusammenfassung

**Zielsetzung: **Die Lehre ärztlicher Gesprächskompetenz schließt die Vermittlung förderlicher Einstellungen ein. Mittels der Communication Skills Attitude Scale (CSAS) werden Einstellungen Medizinstudierender zum Erwerb von kommunikativer Kompetenz erfasst. Ziel der vorliegenden Erhebung ist die Entwicklung einer deutschsprachigen CSAS-Fassung (CSAS-G), um die Einstellungen in einer deutschsprachigen Kohorte zu messen. Ergänzend untersuchten wir den Einfluss demographischer Merkmale auf die Einstellungswerte.

**Methodik:** Wir erstellten die CSAS-G und befragten in unserer Untersuchung 529 Studienteilnehmer aus drei verschiedenen Studienjahren. Wir führten eine explorative wie konfirmatorische Faktorenanalyse durch und verglichen anschließend die Einstellungswerte nach Studienjahr. Ebenso wurde eine multiple Regressionsanalyse berechnet.

**Ergebnisse:** Die konfirmatorische Faktorenanalyse bestätigte das zweifaktorielle System aus der explorativen Faktorenanalyse. Die Studierenden gaben wenig Zustimmung zu negativen Einstellungen und mäßige Zustimmung zu positiven Einstellungen an. Die Einstellungswerte differieren signifikant nach Geschlechtszugehörigkeit.

**Schlussfolgerung: **Die CSAS-G eignet sich zur Einschätzung von Einstellungsausprägungen zu Kommunikationskompetenz in deutschsprachigen Kohorten. Die Medizinstudierenden unserer Studie zeigten grundlegend eine positive Einstellung. Weiterführende Untersuchungen sind erforderlich, um die Einstellungen deutschsprachiger Medizinstudierender zu kommunikativer Kompetenz zu erfassen und zu verstehen.

## Introduction

The physician-patient encounter presents an interpersonal process of highest complexity. The decisive key for the arrangement of a healing patient-doctor relationship is professional communication [1]. With regard to the variety of patients´ needs a physician is supposed to master different communicative challenges. Research has revealed that professional communication can be acquired as ´a series of learned skills´ [2]. Considering communication skills training in medical education, a process towards increasingly detailed educational objectives is observed [3], [4]. To support an adequate communication skills teaching and learning medical education integrates attitude development [5] as underlined by the ongoing professionalism debate [6]. Attitudinal work deepens the learners´ understanding of different communication issues and skills allow the effective transfer into practice [2]. Ajzen as one of the leading attitude scientists states that an `attitude represents a summary evaluation of a psychological object captured in such attribute dimensions as good-bad, harmful-beneficial, pleasant-unpleasant, and likable-dislikable.´ [7]. Consequently attitudes facilitate the adaption of the individual to the environment [8]. Furthermore, according to the Theory of Reasoned Action, attitudes influence behavior [9]. In a qualitative study concerning the Attitude-Social Influence-Self-Efficacy-Model the crucial role of affective components such as emotions with regard to behavior is indicated [10]. Psychosocial attitudes of primary care physicians are related to their communication behaviors as shown by Levinson and Roter [11]. High levels of psychosocial orientation in physicians and medical students are desirable therefore. In contrast Eron [12], [13] noticed a decreasing psychosocial orientation in medical students and shaped the term of dehumanization already in the middle of the twentieth century. This development seems to continue: contemporary studies show lack of increase or even decline in empathy [14], [15] and patient-centeredness [16], [17], [18]. The given trend is mainly attributed to an assumed negative influence of increasing experience in patient interaction. As consequence the third year in medical education seems to be critical for psychosocial orientation since most medical curricula include more direct patient contact after year two [19]. Current research literature on empathy decline reveals distress as second key factor [14]. Research literature offers an instrument asking for attitudes towards communication skills of medical students: the Communication Skills Attitude Scale (CSAS) [20]. Since its development multiple surveys included the original English-speaking or a translated CSAS-version proving the continuing interest in attitudes towards communication skills (see table 1 (Tab. 1)). A German CSAS version was created and applied with German speaking medical students in Hungary [21]. The corresponding publication is written in Hungarian and therefore inaccessible for non-Hungarian speaking audience.

Several studies have examined attitudinal scores before and after a communication skills training [22], [23], [24], [25], [26]. Research using the CSAS shows different patterns of attitude development during medical education, longing from decrease [27], [28], [29] to increase [30]. Anvik and colleagues found stable cognitive attitudes in contrast to decreasing affective attitudes [31], which is similar to the findings of empathy research [32]. Furthermore attitudes towards communication skills appear to be less positive in students with higher levels of state anxiety [33]. 

So far no data about attitudes towards communication skills of German medical students is available. In order to close this gap in educational research a study with the following aims was carried out:

1. Developing a German CSAS version (CSAS-G),
2. validation of CSAS-G with a German cohort,
3. exploring the attitudinal scores in different years of study and
4. investigating the potential impact of demographic factors on attitudinal scores.

## Methods

### Setting

The cohort in this cross-sectional study comprises students in years 1 (n=88), 2 (n=355) and 4 (n=86). Year 1 students´ experience in patient contact depends on individual´s activities before entering medical school. The curriculum in the Leipzig university provides lectures on ´Medical Psychology and Medical Sociology´ without any communication skills training in year 1. Year 2 students attend two communication skills courses. The first part covers 18 hours focusing general communication combined with examples taken from physician-patient communication. Elements such as cooperative communication, active listening [34] and regulation of emotions are integrated. The second training unit covers 21 hours and comprises specific conversation techniques in the physician-patient encounter such as shared-decision making, dealing with taboos, breaking bad news or life-style counseling [35], [36], [37]. Year 4 students have comparatively much patient contact due to a regular bed-side teaching and compulsory clerkship. In the clinical part no communication skills training is offered. 

#### Data collection

##### 1. Procedure

The original CSAS was translated into German using standard forward-backward procedure supported by a native English speaker. The first author contacted Charlotte Rees via email for detailed clarification of item meaning. The two steps of translation process were performed repeatedly and supplemented by a pretest until a satisfying German version close to the English original was developed (see table 2 (Tab. 2), translated CSAS is part of the German version of this article). 

Since students from three different years of medical school with different access to communication skills training were involved, communication skills training related wordings were adapted. Year 1 students have had no contact to communication skills training when answering the questionnaire. Therefore item 12 „Learning communication skills is fun.“ was modified into „Learning communication skills will be fun.“ No adaption of wording was indicated for year two students that filled out the CSAS-G shortly after they had attended the communication skills training. 

During courses participants had been invited to participate voluntarily. They were informed about anonymous data analysis. The questionnaires were passed to the students by one of the authors giving a short instruction without mentioning the aim of the study. Sensitive personal information was not required and no kind of experiment was part of the study. Therefore ethical approval was not necessary considering rules of ethics commission of the University of Leipzig.

##### 2. Instrument

Students of our subject group completed the CSAS-G together with a demographic questionnaire including year of study, age and gender. The CSAS itself consists of 26 items with statements towards learning, teaching and using communication skills. Responders choose along a five-point Likert scale that ranges from 1 (strongly disagree) to 5 (strongly agree). Therefore higher values express stronger attitudes. According to the original validation the CSAS measures attitudes along two dimensions:

1. Positive attitude scale (PAS) and
2. Negative attitude scale (NAS).

##### 3. Statistics

Data analysis was performed using the Statistical Package for Social Sciences (SPSS version 18.0). An explorative factor analysis with direct oblimin rotation was conducted and Cronbach´s a for each factor was calculated leading to a reduction in item numbers of the two subscales. Confirmatory factor analysis to test the factorial structure was performed for the reduced subscales and the original subscales additionally. AMOS 20 was used as statistical program for this purpose. The confirmatory factor analyses were compared to each other based on the following model fit indices: the minimum discrepancy, divided by its degrees of freedom (CMIN/DF); the goodness-of-fit-index (GFI); the normed-fit-index (NFI); the comparative-fit-index (CFI); the Tucker-Lewis-Index (TLI); the root mean square error of approximation (RMSEA); and the Akaike Information Criterion (AIC). The ratio CMIN/DF should be possibly small for a satisfactory model fit [38], [39]. GFI ought to range between 0.97 and 1 and NFI is ideally higher than 0.95 [39]. A good model fit is indicated by values of CFI and TLI close to 0.95 or even higher [39], [40]. RMSEA should be 0.05 or smaller. A descriptive indicator of the badness of fit is provided by the AIC. It allows comparisons of two varying models whereby the lower AIC belongs to the preferable model [38], [39]. In order to test each model we used covariance matrices and the maximum likelihood method approach. Attitude scores were calculated by dividing the sum of item scores of each subscale by the number of items per subscale according to factor analysis result. Correlation between attitude scores and demographic characteristics were studied using Pearson´s correlation coefficient. Normality was assessed by the help of the Kolmogorov-Smirnov test, which indicated a missing normal distribution. Therefore we used the non-parametric Kruskal-Wallis-H-test for comparison of CSAS scores of different study years. As a result significant rank differences were revealed. Assuming that significant non-parametric test results justify using a parametric test without normal distribution, we conducted t-tests to study differences of male and female CSAS scores. Comparisons between CSAS scores of the three different years of study were based on single factor variance analyses with post-hoc Scheffé-test. To determine group differences the following levels of significance were applied: *p<0.05, **p<0.01 and ***p<0.001. Effect sizes (d) were calculated for significant differences in attitude scores because of varying subcohort sizes. Effect sizes d>0.50 are interpreted as large, 0.50>d>0.30 as medium, 0.30>d>0.10 as small and d<0.10 as trivial [41]. 

## Results

### Demographic results

529 questionnaires have been completed satisfactorily. The subjects´ age ranged from 19 to 47 years (mean age= 26 years). Female participants build the majority in the sample (64%) in comparison to male participants (36%) (see table 3 (Tab. 3)). The gender distribution of this cohort corresponds to the predominantly female fraction of medical students in Germany [42].

#### Validation of CSAS-G

The Kaiser-Meyer-Olkin measure was 0.899 and a positive p-value of <0.001 was revealed by Bartlett´s test of sphericity, both results showing the adequacy for conducting factor analysis. The initial explorative factor analysis showed seven factors with eigenvalues larger than 1, explaining 58% of variance. The original validation [20] offered a two-factor solution with 13 items per subscale. We tried to replicate this in a second explorative factor analysis with determination of two factors. The derived factors were not identical to those in their study. Nevertheless, we decided to follow the two subscales´ system because of the qualitative basement provided by Rees and colleagues [43], [44], [45]. Items were assigned to one factor, if they load at least 0.28 on one factor (see table 4 (Tab. 4)). Items 01, 03, 08, 13, 18, 20 and 22 were excluded due to minimal or ambiguous loading on one factor. As a result the subscale NAS in our sample contains 7 items (Cronbach´s α=0.838) and the subscale PAS consists of 12 items (Cronbach´s α=0.864). The Pearson correlation coefficient for PAS score and NAS score in the total study sample is r=-0.49 (p<0.001) indicating a converse linear relationship between PAS score and NAS score which supports the founding idea of the original subscale structure: a low PAS score is associated with a high NAS score and reversely. 

Subsequently we calculated the confirmatory factor analysis. The results for the original subscales were less satisfactory. The model for the CSAS-G was estimated in a second step. The reduced subscale structure was adopted from the results of the second explorative factor analysis. We found an insufficient model fit. Therefore we gradually allowed correlations between single item-influencing error indices by considering the respectively highest modification indices. As a result an assumable model was won. Therefore we consider our two factor model of explorative factor analysis as confirmed. Consequently we used the CSAS-G subscales as described in our explorative factor analysis for further calculations.

#### Attitudinal scores 

The attitudinal scores of both subscales in relation to year of study are presented in table 5 (Tab. 5).

Negative attitudes towards communication skills slightly increase from year 1 to year 2. Negative attitudes are more pronounced in year 4 in comparison to year 1. There was a significant difference between year 2 and year 4. With regard to mean scores measured along the PAS subscale, a significant reduction in positive attitude levels occurs from year 1 to year 2, followed by rising levels from year 2 to year 4. 

In the complete study sample female students show lower NAS scores than male students (mean=2.32) (p=0.000; d=0.48) and higher PAS scores (mean=3.12) than male students (mean=2.80) (p=0.000; d=0.38). Calculating the correlation coefficients for age and subscale scores identified no clear correlation (PAS score/age: r=-0.49 (p=0.43); NAS score/age: r=0.09 (p<0.05)). With respect to gender a correlation was deducted (PAS score/gender: r=0.21 (p<0.001); NAS score/gender: r=-0.21 (p<0.001)). 

#### Regression analysis

Multiple regression (see table 6 (Tab. 6)) was conducted progressively. In the first step a low significance for age is detected referring to the NAS score. However, in the second step adding gender, the significance of age is cancelled whereas the impact of gender on NAS and PAS score is highly significant. We repeated the procedure using year of study instead of age without finding significant different results. As consequence regression analysis reveals that gender has some bearing on CSAS scores. In general, the contribution of the regression analysis is limited as indicated by R²=0.044/0.041.

## Discussion and conclusion

### Discussion 

(1) First purpose of this study was to translate the English CSAS (CSAS-E) into German. In our study we used a forward-backward procedure for translation to win CSAS-G. Any translational procedure affects the study results. It is known that even slight changes in item wording influence understanding of interviewees and measurement [46]. The potential impact of translation is magnified by the modifications of wording for study year 1 (see Procedure), which therefore presents a study limitation. In our case we consider adaption of wording as necessary to raise the understanding for study participants of study year 1. Despite the translation-related effects the original CSAS contains wordings that stand in contrast to general recommendations for questionnaire construction. Items should be phrased without suggestion or negation for best possible interviewee understanding [47]. CSAS wordings like ´Communication skills teaching would have a better image if it sounded more like a science subject.´ (Item 17) or ´I don´t need good communication skills to be a doctor.´ (Item 19) can reduce survey participants´ understanding.

(2) Second aim of this survey was to validate the CSAS-G. The two subscales of the CSAS-G are reliable after elimination of seven items. Confirmatory analysis approved this subscale system. To reach a satisfactory model fit stepwise modification was necessary. This fact points out a limited validity of the questionnaire. A validity check of good quality was not feasible due to missing German-speaking instruments that cover medical students´ attitudes towards communication skills.

Rees and colleagues [20] initially describe a six-factor solution but decided to stick to a two-subscale system. In multiple validations this two-factor structure was confirmed, even though in some cases different items were assigned to the two subscales. Three other studies clearly describe elimination of items [48], [49], [50]. Items that have been eliminated repeatedly in several studies were not identified. Beside the two-factor solution of the CSAS-E, other subscale systems were developed (see table 1 (Tab. 1)). The factor solution for our sample does not replicate any of the other subscale structures described in research literature. 

The variety of validation results can be ascribed to translation. Considering the different cultures and languages various factor-models can be evaluated as acceptable. Similar findings are known from other questionnaires that have been translated. 

Another possible reason for varying CSAS factor solutions can be seen in selection of interviewees. Whereas the original CSAS was designed for medical undergraduates, research literature presents studies with psychology students [25], dental students [51], [52], teachers [53], dietetic students [54] and nursing students [50]. In view of known factor analytic difficulties and critical item wordings some authors deduce a need for a modification of the original CSAS [48], [50]. 

(3) The third aim of our study was to explore the attitudinal scores of medical undergraduates towards communication. Our results show low levels of negative attitudes and moderate levels of positive attitudes. We conclude that students in our cohort like communication therefore. In our study voluntary participation could mean that surveyed students are generally more interested in communication. Comparison of mean scores and year of study in our cohort conveys the following picture: the NAS scores increase significantly and PAS scores decrease. According to significant mean differences illustrating a negative trend, one may subsume a decline of attitude towards communication skills in conclusion. With regard to absolute means and their development, it is questionable whether this decline truly impacts attitudes. No influential change along the Likert-scale can be found with PAS means around a score of 3 and NAS means around 2. In our study findings no evidence of a decline of practical implication value is present [55]. Research literature offers only single other works that show similar results [56], [57]. These conclusions with regard to attitudinal development have only limited expressiveness because of the cross-sectional study design. To truly measure the course of attitudes during medical school a longitudinal survey is indispensable. 

Another limitation is given by the unequal sample sizes per study year. The majority of the cohort is built by year 2 students. To avoid sample size-related bias we calculated additionally the effect sizes which confirmed the significant mean differences. 

As known from another CSAS study women show more favorable attitudes [45]. This tendency is also present in our study findings. Two Asian CSAS studies reveal no significant differences between female and male attitude scores [58], [59]. An alternative study result was found in a UK cohort where an increase in male empathy scores in contrast to decreasing female scores was detected [60]. 

#### Conclusion and practice implication

In medical education exists sustained interest in attitudinal research. The CSAS is a helpful instrument to evaluate students´ attitudes towards communication skills. Translation delivered the CSAS-G which proved to be reliable in our pilot study. In principle CSAS-G is suitable for German-speaking medical education purposes. Under certain circumstances rephrasing of specific items is recommended for closer adaption to the respective study object. Beside the use of the translated CSAS version, no accompanying qualitative evaluation of the students´ views towards communication skills learning has been conducted. In order to develop a German version considering special thoughts of German students, further research will be beneficial [61]. 

We saw difficulties in factor analytic verification that are already known from other CSAS-studies, even from the original validation of CSAS-E. Up to now a study comparing the different factor-models is missing. Further studies are necessary to examine validity and test-retest-reliability. So far it is questionable whether the construction of negative and positive attitudes towards communication skills aligns reality.

The students in our study seem to like communication. For a deeper understanding of attitude development future research including a longitudinal design is necessary. 

## Acknowledgements

We thank our study participants. Thanks to Prof. Charlotte Rees for allowing and supporting us to translate the CSAS and Prof. Andreas Hinz for his revision suggestions. 

## Competing interests

The authors declare that they have no competing interests.

## Figures and Tables

**Table 1:**
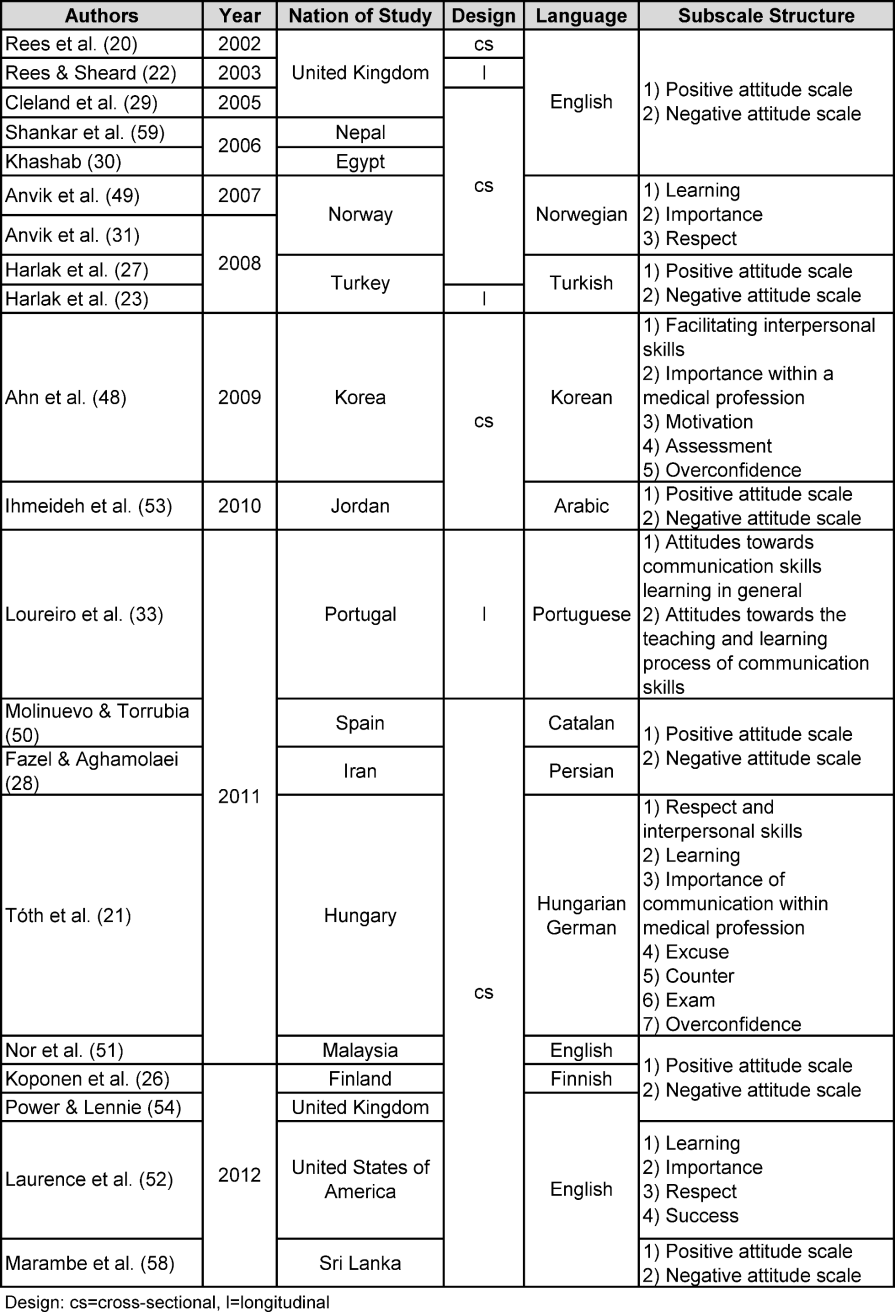
Overview CSAS surveys (Reference numbers as cited in the reference list).

**Table 2:**
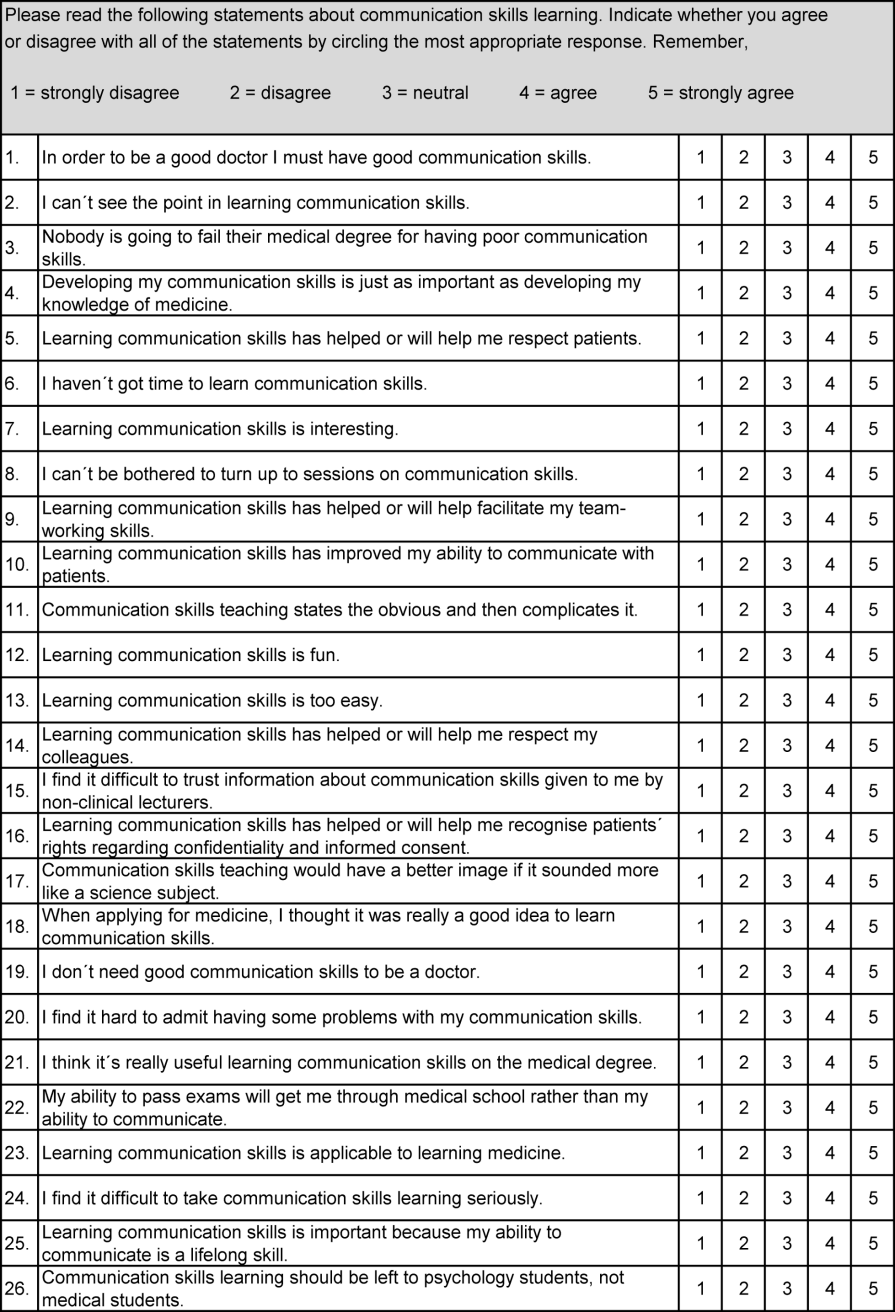
Communication Skills Attitude Scale (CSAS)

**Table 3:**
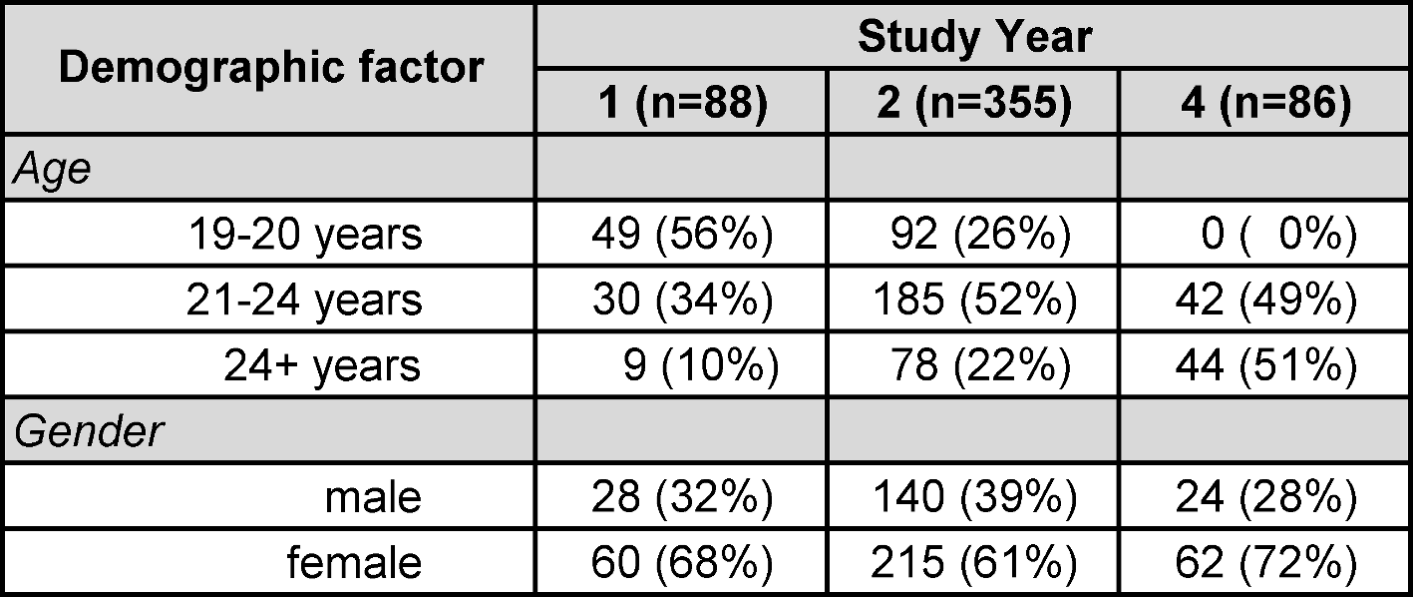
Demographic characteristics of the study sample in absolute numbers and proportions (in parentheses).

**Table 4:**
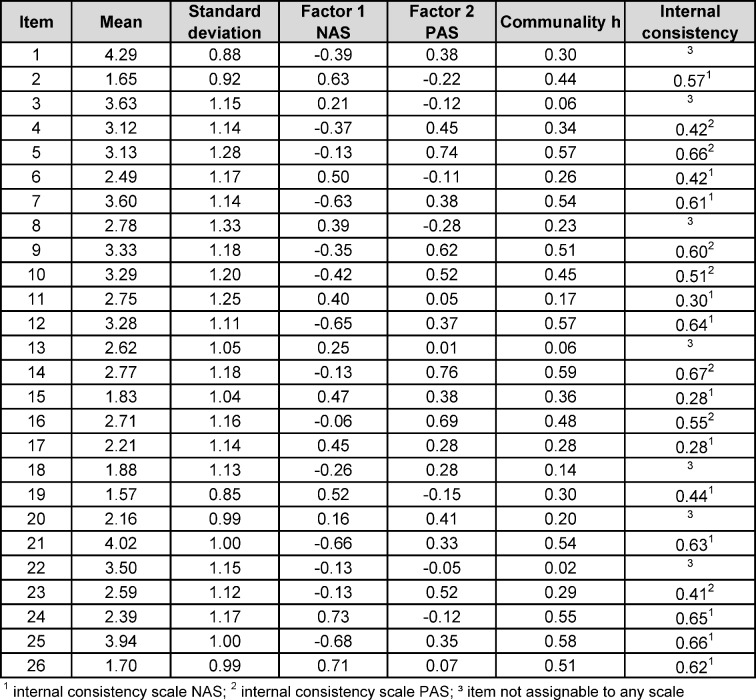
Rotated component matrix.

**Table 5:**
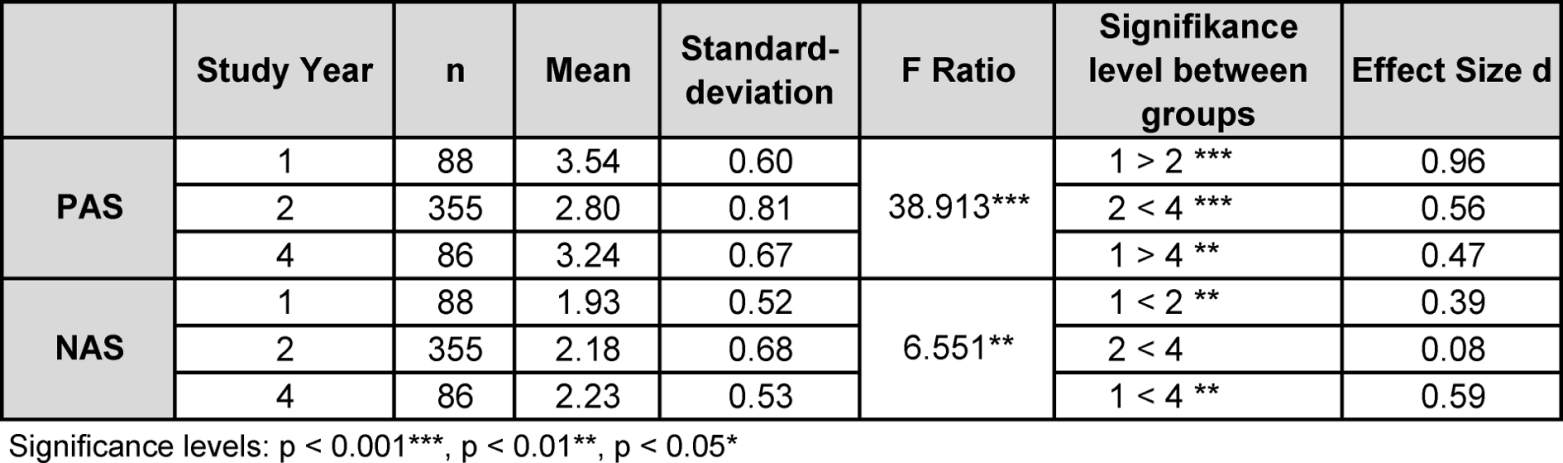
Single factor variance analyses: Comparison of attitudinal scores in relation to year of study.

**Table 6:**
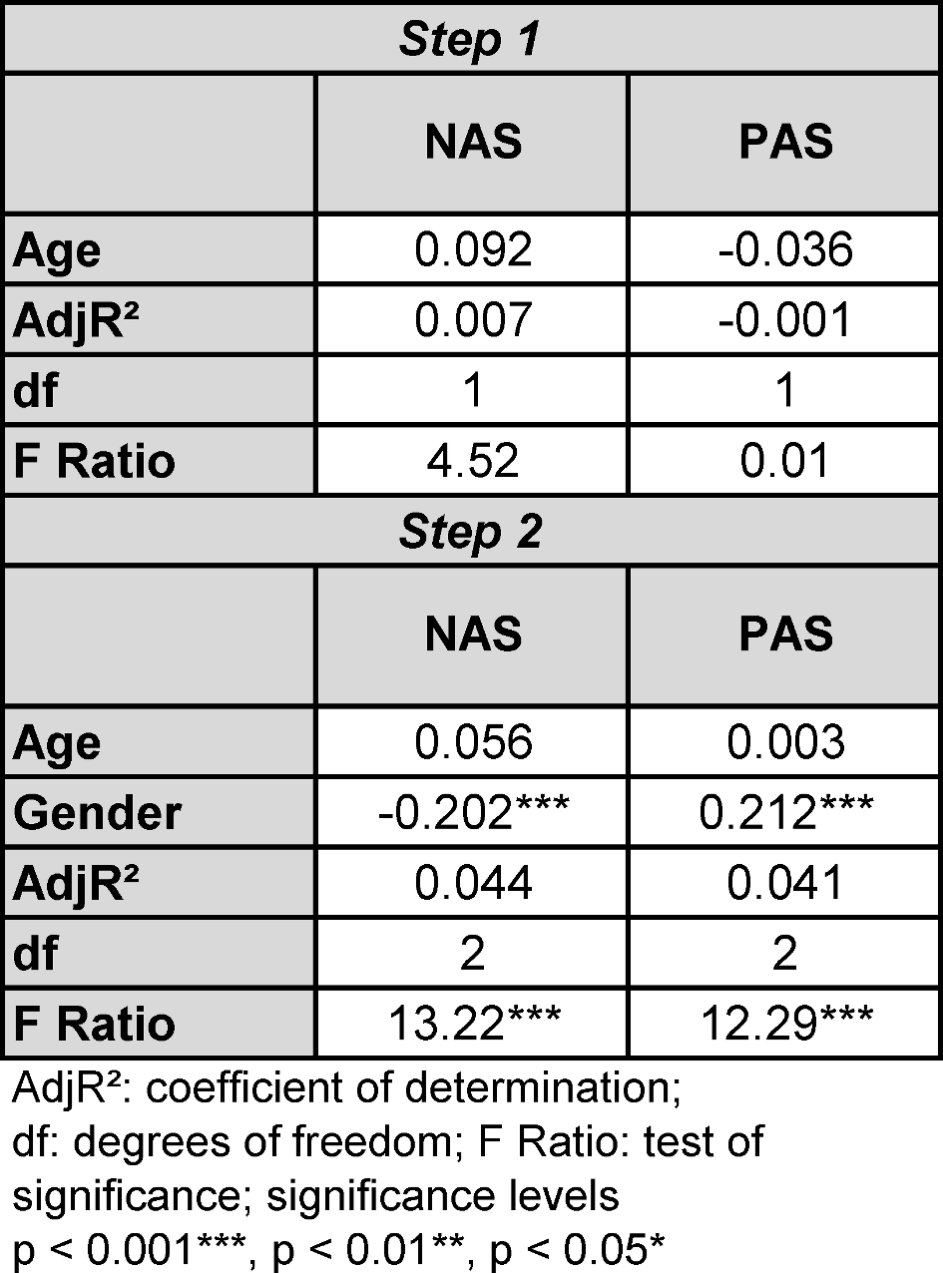
Multiple regression.
